# CCL24 contributes to HCC malignancy via RhoB- VEGFA-VEGFR2 angiogenesis pathway and indicates poor prognosis

**DOI:** 10.18632/oncotarget.14095

**Published:** 2016-11-24

**Authors:** Lei Jin, Wei-Ren Liu, Meng-Xin Tian, Xi-Fei Jiang, Han Wang, Pei-Yun Zhou, Zhen-Bin Ding, Yuan-Fei Peng, Zhi Dai, Shuang-Jian Qiu, Jian Zhou, Jia Fan, Ying-Hong Shi

**Affiliations:** ^1^ Department of Liver Surgery, Liver Cancer Institute, Zhongshan Hospital, Fudan University, Key Laboratory of Carcinogenesis and Cancer Invasion of Ministry of Education, Shanghai, China; ^2^ Institutes of Biomedical Sciences, Fudan University, Shanghai, People's Republic of China

**Keywords:** CCL24, HCC, RhoB, VEGFA, prognosis

## Abstract

CCL24 is one chemotactic factor extensively studied in airway inflammation and colorectal cancer but less studied in hepatocellular carcinoma (HCC) retrospectively. So HCC tissue microarray (TMA) was used to estimate relationship between CCL24 and prognosis, cell experiments were conducted to study its influence for HCC cell biological behavior. CCL24 was injected to nude mice to monitor tumor formation and pulmonary metastasis; qRT-PCR, western blot and Immunohistochemistry were used to explore potential mechanism. CCL24 plays roles in target cells via its downstream CCR3, or it is regulated by Type 2 helper T cells (Th2 cell) factors, so immune related experiments were conducted. Meanwhile, Rho GTPase family have close relation not only with T cell priming, but with neovascularization; CCL24 contributes to neovascularization in age-related macular degeneration via CCR3, so Rho GTPase family, Th2 cell factors, Human Umbilical Vein Endothelial Cells were used to uncover their trafficking. Ultimate validation was confirmed by small interfering RNA. Results showed CCL24 expression was higher in caner tissues than adjacent normal tissues, it could contribute to proliferation, migration, and invasion in HCCs, could accelerate pulmonary metastasis, promote HUVECs tube formation. Th2 cell factors were irrelevant with CCL24 in HCCs; and RhoB, VEGFA, and VEGFR2 correlated with CCL24 in both mRNA and protein level. Downstream RhoB-VEGFA signaling pathway was validated by siRhoB and siVEGFA inhibition. In a word, CCL24 contributes to HCC malignancy via RhoB-VEGFA-VEGFR2 angiogenesis pathway and indicates poor prognosis, which urges us to study further CCL24 effects on diagnosis and potential therapy for HCC.

## INTRODUCTION

Hepatocellular carcinoma (HCC) is the second leading cause of cancer-related deaths worldwide with an ascending trend in recent years for its various pathogenesis [1], including Hepatitis B Virus (HBV) or Hepatitis C virus (HCV) infection [2], alcohol abuse [3], metabolic syndromes [4], etc. In spite of some molecular markers being approved for clinical applications, specific molecular mechanism of HCC has not yet been clarified [5, 6]. Chemokines are cytokines that stimulate pro-inflammatory activity by eliciting the chemotactic migration of certain cells to target sites to cause inflammatory conditions [7]. Their infiltration affected the balance of relevant immune cells both within and outside of their target organs, then mediated tumor initiation, metastasis, and relapse by chemokines’ autocrine or paracrine manners [8].

CCL24, known as eotaxin-2, MPIF-2, or Ckβ-6, had been studied in allergies and eosinophilic esophagitis for many years since its discovery in 1997 [9–11]. CCL24 was expressed by cytokeratin^+^ epithelial cells, CD31^+^ endothelial cells, and CD68^+^ macrophages [12], especially in asthma, CCL24 facilitated eosinophil migrating into lungs by upregulating adhesion ability to endothelial cells [13]. Meanwhile, CCL24 has been extensively studied in colorectal tumors these years. One original study revealed interleukin-4 (IL-4) and interleukin-13 (IL-13) could stimulate overexpression of CCL24 in colorectal cancer cell, and CCL24 expression was strongly associated with poor prognosis for patients with colorectal cancer [14]; another research further disclosed the quantitative difference of CCL24 between glandular cells and colorectal neoplasms [15]. As the downstream of CCL24, CC chemokine receptor 3 (CCR3) was also involved in many cancer. Miyagaki found that CCR3 was associated with anaplastic large cell lymphoma (ALCL) cells via ERK1/2 activation [16]; CCR3 played a role in the recruitment and retention of CD30^+^ malignant T cells to the skin in skin-specific cutaneous T-cell lymphoma (CTCL) [17]. Certainly, the study of cancer-related inflammation enhancing tumor cell survival, proliferation, and metastasis has flourished over the last few decades, however, the role of CCL24 in HCC has not been investigated, considering liver is homologous with the gut in embryonic development, there was an urgent need to investigate CCL24 expression in HCC as well as its potential contribution to the inflammatory microenvironment in HCC [7].

In our study, we initially investigated the expression of CCL24 in fresh HCC tissues and HCC cell (HCCs) lines, the prognosis role of CCL24 was also analyzed. Biological behaviors such as proliferation, migration, and metastasis of HCCs lines were induced by CCL24 overexpression or CCL24 knockdown. *In vivo* tumorigenesis in nude mice was also analyzed by CCL24 interference. Subsequent CCR3, Type 2 helper T cells (Th2 cell) factors, Rho GTPase family, VEGFA, etc. were used to explore their relevance with HCC, qRT-PCR, western blot and Immunohistochemistry were used to explore potential mechanism, ultimate speculation or conclusion was investigated by siRNA validation.

## RESULTS

### CCL24 was upregulated in HCC tissues and was correlated with poor prognosis in HCC patients

**Figure 1:**
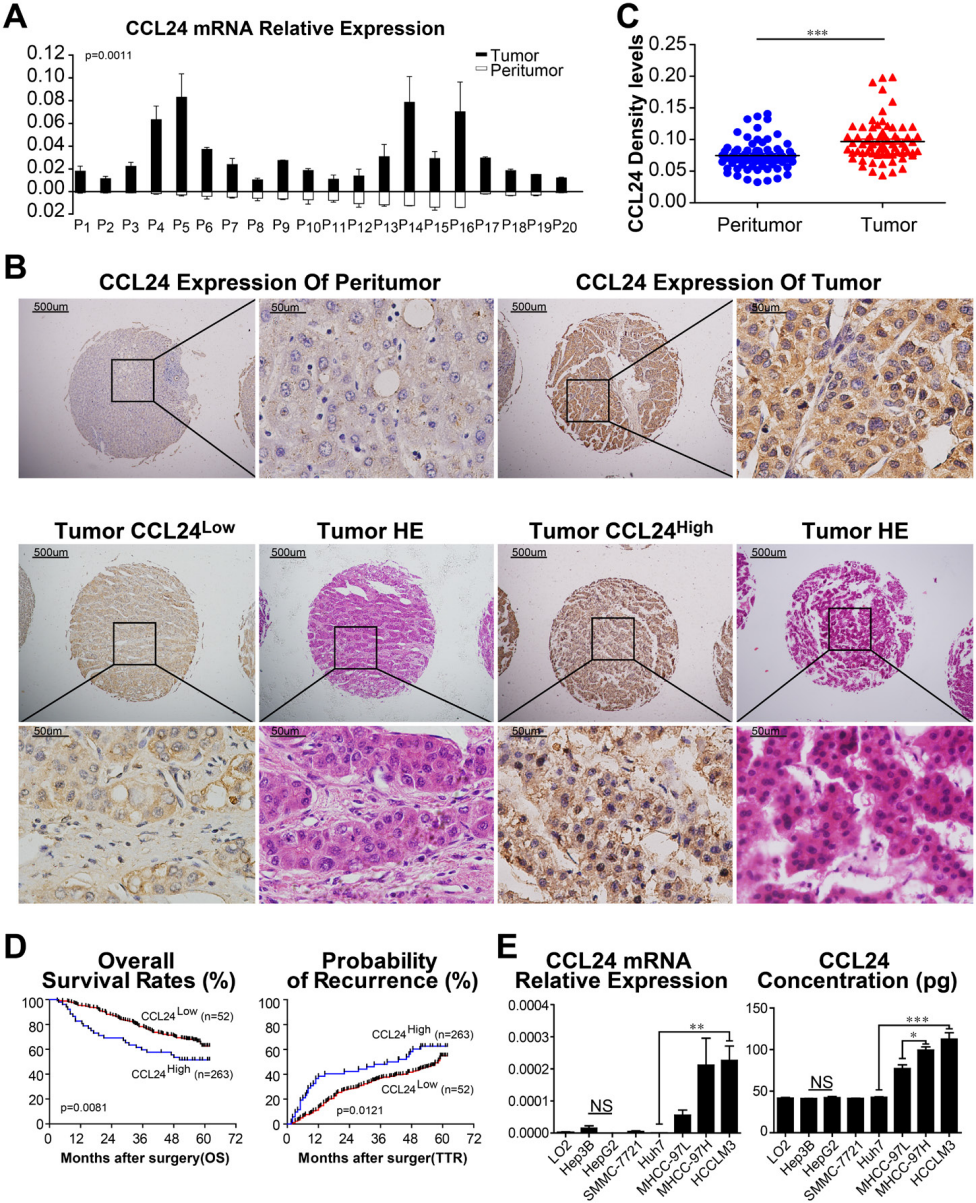
CCL24 expression in HCC tissues and HCC cell lines **A**. CCL24 mRNA expression in 20 fresh HCC tumors and adjacent normal tissues. **B**. Representative photomicrographs of peritumor and tumor tissues showed CCL24 expression (brown staining in the cytoplasm of cells). Scale bar, 40×, 500 um; 400×, 500 um. **C**. Density analysis showed statistical significance of CCL24 expression of 70 cases of patients in TMA samples. **D**. Prognostic values of CCL24 expression using Kaplan-Meier analysis. **E**. qRT-PCR analysis and ELISAs of CCL24 expression in normal liver cell (L02) and seven HCC cell lines (Hep3B, HepG2, SMMC-7721, Huh7, MHCC-97L, MHCC-97H, HCCLM3). Data shown were means (±SD) from three independent experiments. *P< 0.05, **P< 0.01, ***P<0.001.

First, we compared mRNA levels in 20 fresh HCC tissues with their paired adjacent normal tissues. Results revealed that CCL24 expression was remarkably higher in HCC tissue than adjacent normal tissues (p=0.0011; Figure 1A). We next examined CCL24 expression by TMA to explore its prognostic value in HCC patients. The immunohistochemistry experiment showed that CCL24 was dominantly expressed in the cytoplasm of HCC tissues in contrast to adjacent normal tissues (Figure 1B). Density analysis of TMA also revealed that CCL24 expression was clearly more prominent in HCC tissues than in the adjacent normal tissues (p<0.0001; Figure 1B, 1C). Upon further analysis of the clinical characteristics of 315 HCC patients, we found high CCL24 expression was significantly correlated with increased age (p=0.009), positive HBsAg (p=0.026), and larger tumor size (p=0.036). Other clinical characteristics, including sex, α-fetoprotein (AFP), γ-GT, liver cirrhosis, tumor number, tumor encapsulation, microvascular invasion, tumor differentiation, tumor-nodes-metastasis (TNM) stage, and Barcelona Clinic Liver Cancer (BCLC) stage were not directly correlated with the expression of CCL24 (Table 1).

**Table 1:** Clinical and Demographic Characteristics of 315 HCC Patients

Clinicopathological indexes		315 HCC Patients		P†
		CCL24^LOW^ (n=263)	CCL24^HIGH^ (n=52)	
Sex	Female	44	8	0.811
	Male	219	44	
Age(year)	≤50	112	12	**0.009**^†^
	>50	151	40	
HBsAg	Negative	34	13	**0.026**^†^
	Positive	229	39	
HCV	Negative	263	50	**0.027**^†^
	Positive	0	2	
AFP	≤20	101	26	0.119
	>20	162	26	
γ-GT(U/L)	≤54	144	26	0.530
	>54	119	26	
Liver cirrhosis	No	50	12	0.500
	Yes	213	40	
Tumor number	Single	223	44	0.974
	Multiple	40	8	
Tumor size(cm)	≤5	172	26	**0.036**^†^
	>5	91	26	
Tumor encapsulation	complete	146	33	0.290
	none	117	19	
Microvascular invasion	absence	185	36	0.873
	present	78	16	
Tumor differentiation	I+II	201	44	0.194
	III+IV	62	8	
TNM stage	I	160	32	0.924
	II+III	103	20	
BCLC stage	0+A	159	24	0.056
	B+C	104	28	

Abbreviations: HCC, hepatocellular carcinoma; HBsAg, hepatitis B surface antigen; AFP, α-fetoprotein; γ-GT,γ-glutamyl transpeptadase; TNM, tumor-nodes-metastasis; BCLC, Barcelona Clinic Liver Cancer. ^†^P-value < 0.05 was considered statistically significant. P-values were calculated using the Pearson chi-square test.

By the last follow-up, in December 2011, 49.8% (157/315) of the patients had suffered recurrence and 35.6% (112/315) had died. The 1-, 3-, and 5-year overall Survival (OS) rates in the CCL24^Low^ group were 95.4%, 78.3%, and 62.6%, respectively, which were higher than those of the CCL24^High^ group, which were 82.7%, 59.6%, and 51.5%, respectively. In addition, the cumulative recurrence rates of the CCL24^Low^ group were 61.5%, 51.9%, and 37.4%, respectively, which were significantly lower than those of the CCL24^High^ group, which were 86.7%, 63.1%, and 45%, respectively (Figure 1D). The median lifetime OS and cumulative recurrence of the CCL24^Low^ group were both higher than those of the CCL24^High^ group (60 months vs. 57.7 months and 57.2 months vs. 38.3 months, respectively). Meanwhile, the OS and TTR of the CCL24^Low^ group were both longer than those of the CCL24^High^ group (p=0.0081, p=0.0121; Figure 1D). Further investigations revealed the prognostic value of CCL24 expression in HCCs with AFP > 20 ng/mL, without liver cirrhosis, within a solitary tumor, microvascular invasion, and TNM stage II+III in the HCC patient subgroups (Supplementary Figure S1A).

In univariate analysis, γ-GT, tumor number, tumor size, microvascular invasion, TNM stage, BCLC stage and CCL24 level were associated with OS, whereas, γ-GT, liver cirrhosis, tumor number, tumor size, microvascular invasion, TNM stage, BCLC stage and CCL24 level were associated with TTR. In multivariate analysis, γ-GT, tumor size, TNM stage and CCL24 level were associated with OS, whereas, γ-GT, liver cirrhosis, tumor number, microvascular invasion, BCLC stage and CCL24 level were associated with TTR. Univariate and multivariate analyses demonstrated that CCL24 expression in tumor cells served as an independent risk factor for both OS and TTR (*P* = 0.013, HR = 1.783; P = 0.012, HR = 1.666; Table 2).

**Table 2:** Univariate and Multivariate Analyses of Factors Associated with Survival and Recurrence of HCC patients

Factor	OS				TTR			
	Univariate *P*	Multivariate			Univariate *P*	Multivariate		
		HR	95% CI	*P*^†^		HR	95% CI	*P*†
Sex (female vs. male)	0.999			NA	0.134			NA
Age, years (≤50 vs. >50)	0.175			NA	0.207			NA
HBsAg (negative vs. positive)	0.887			NA	0.361			NA
AFP, ng/ml (≤20 vs. >20)	0.161			NA	0.407			NA
γ-GT, U/L (≤54 vs. >54)	**0.004**^†^	1.552	1.056-2.281	**0.025**^†^	**0.003**^†^	1.476	1.069-2.036	**0.018**^†^
Liver cirrhosis (no vs. yes)	0.115			NA	**0.009**^†^	1.879	1.165-3.029	**0.010**^†^
Tumor number (single vs. multiple)	**<0.001**^†^			NS	**<0.001**^†^	1.578	1.049-2.373	**0.028**^†^
Tumor size, cm (≤5 vs. >5)	**<0.001**^†^	1.618	1.097-2.387	**0.015**^†^	**0.002**^†^			NS
Tumor encapsulation (complete vs. none)	0.348			NA	0.349			NA
Microvascular invasion (no vs. yes)	**0.014**^†^			NS	**0.005**^†^	1.458	1.038-2.048	**0.030**^†^
Tumor differentiation (I-II vs. III-IV)	0.242			NA	0.174			NA
TNM stage (I vs. II III)	**<0.001**^†^	1.595	1.010-2.519	**0.045**^†^	**<0.001**^†^			NS
BCLC stage (0/A vs. B/C)	**<0.001**^†^			NS	**<0.001**^†^	1.448	1.023-2.050	**0.037**^†^
CCL24 level (low vs. high)	**0.009**^†^	1.783	1.130-2.812	0**.013**^†^	**0.013**^†^	1.666	1.121-2.475	**0.012**^†^

Abbreviations: OS, overall survival; TTR time to recurrence; AFP: α-fetoprotein; γ-GT, γ-glutamyl transferase; TNM, tumor-nodes-metastasis; BCLC, Barcelona Clinic Liver Cancer; HR, hazard ratio;

CI, confidential interval; NA, not adopted; NS, not significant. *^†^P-value < 0.05 was considered statistically significant*. Cox proportional hazards regression model.

### CCL24 expression was associated with the metastatic potential of HCC cell lines and promoted proliferation, migration, and invasion

**Figure 2:**
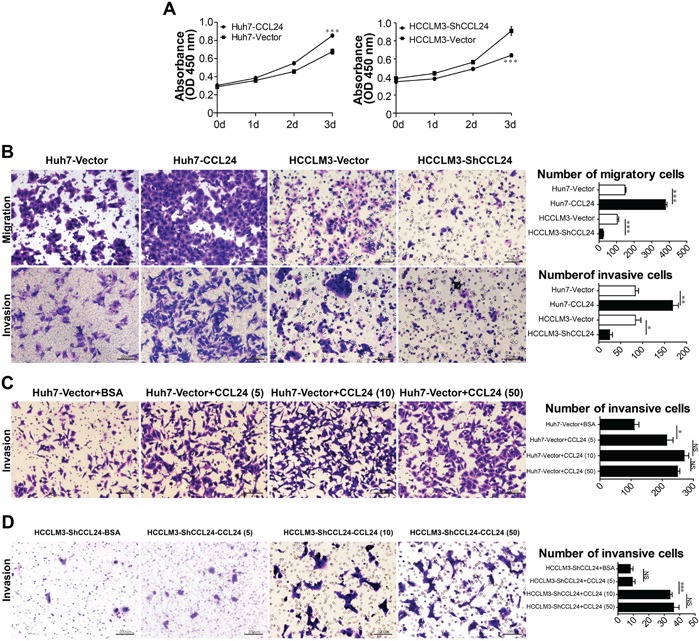
Characterization of CCL24 expression, proliferation, migration, and invasion ability in HCC cells **A**. Cell proliferation was detected by CCK8 assay. **B**. The migration and invasion of cancer cells were measured by transwell assays after being stably transfected lentivirus. Scale bar, 100×, 200 um. **C-D**. The invasion of cancer cells were measured by transwell assays after adding different concentration of CCL24 IgG/BSA (respectively: 5 ng/ml, 10 ng/ml, 50 ng/ml). Scale bar, 100×, 200 um. Data shown were means (±SD) from three independent experiments. *P< 0.05, **P< 0.01, ***P<0.001.

Based on the observation of CCL24 in the clinical analysis, we next investigated the mRNA and protein levels of CCL24 in HCC cell lines. qRT-PCR and ELISA revealed that the mRNA and protein levels of CCL24 were significantly higher in highly metastatic HCC cell lines (MHCC-97H and HCCLM3) compared to less metastatic HCC cell lines (Hep3B, HepG2, SMMC-7721, and Huh7) and a normal liver cell line (L02) (Huh7 vs. HCCLM3: p=0.0074; p=0.0008; Figure 1E). Proliferation, migration, and invasion of HCC cells were explored by upregulation of CCL24 expression in Huh7 cells and knockdown of CCL24 in HCCLM3 cells (Figure 2; Supplementary Figure S2). We found that proliferation of Huh7-CCL24 cells was significantly higher than that of Huh7-Vector cells (p<0.0001; Figure 2A), and the proliferation of HCCLM3 cells was inhibited by CCL24 knockdown (p=0.0008; Figure 2A). In the *in vitro* migratory and invasive experiments, the number of migratory and invasive cells in the Huh7-CCL24 group was significantly higher than that in the control group (378±9.5 vs. 148±5.7, p< 0.001; 167.7±13.6 vs. 83±7.5, p=0.0055; Figure 2B), whereas the number of migratory and invasive cells in the HCCLM3-ShCCL24 group was significantly decreased compared to the control cells (22.7±3.5 vs. 103.3±8.6, p=0.001; 24.7±6.4 vs. 83.3±12.6, p=0.0141; Figure 2B). To gain insight into the effect of CCL24 on HCC cell invasion, we added CCL24 IgG/BSA to Huh7-Vector and HCCLM3-ShCCL24 culture supernatants at various concentrations as previously described [14]. We found that the number of invasive cells was dose-dependent by microscopic examination (Figure 2C, 2D). The Huh7-Vector group was more sensitive than the HCCLM3-ShCCL24 group when cultured with CCL24 at 5 ng/ml (214±20.8 vs. 111±14.1, p=0.0149; Figure 2C), while the HCCLM3-ShCCL24 group showed a statistically significant difference when the CCL24 concentration was at 10 ng/ml (33.7±1.5 vs. 9.3±1.8, p=0.0004, Figure 2D). However, their invasive ability did not increase in concert with CCL24 concentration (Figure 2C, 2D).

### CCL24 contributed to progression and metastasis of HCC *in vivo*

**Figure 3:**
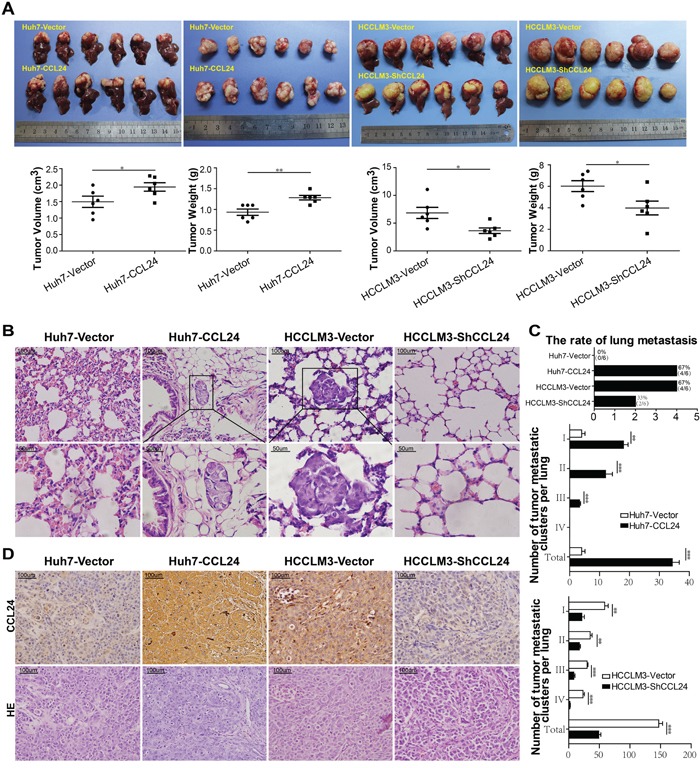
CCL24 promotes HCC progression in a xenograft nude mice model **A**. Liver bearing tumors formed by implanted cells with different expression levels of CCL24. Weights and tumor volume of the tumors in livers of nude mice were measured. Error bar indicates standard deviation (n=6). **B**. H&E-stained images of metastatic nodules in lungs from all groups with magnification of the selected areas. Scale bar, 200×, 100 um; 400×, 50 um. **C**. The counts of tumor rates, metastatic clusters in lung from all xenograft nude mice groups. **D**. Representative images from tumor serial sections stained with CCL24 by immunohistochemistry. Scale bar, 200×, 100 um. *P< 0.05, **P< 0.01, ***P<0.001.

After subcutaneous injection of these cell lines into nude mice, all of the groups successfully formed liver tumors. The tumor volume of the Huh7-CCL24-derived xenografts was 1.9±0.1 cm^3^, which was larger than that of the xenografts derived from Huh7-Vector cells (1.5±0.2 cm^3^, p=0.0437; Figure 3A). The tumor weight of the Huh7-CCL24-derived xenografts was 1.3±0.1 g, which was higher than that of the Huh7-Vector cells (0.9±0.1 g, p=0.0038; Figure 3A). In addition, the tumor volume and weight of the HCCLM3-Vector-derived xenografts were 6.8±1.0 cm^3^ and 6.0±0.5g, respectively, which were clearly larger and higher than those of the HCCLM3-ShCCL24 cells (3.6±0.5 cm^3^, p=0.0166; 4.0±0.6 g, p=0.0316; Figure 3A). In the HCCLM3-Vector and Huh7-CCL24 nude mice, the incidences of pulmonary metastasis were both 67% (4/6), while the incidences of pulmonary metastasis in the Huh7-Vector and HCCLM3-ShCCL24 groups were 0% (0/6) and 33% (2/6), respectively (Figure 3B, 3C). Meanwhile, the CCL24 expression levels measured by immunohistochemistry in different xenograft tumors were also in line with the previous outcomes of cell lines after lentivirus interference (Figure 3D).

### CCL24 enhanced HUVEC tube formation and contributed to HCC malignancy via RhoB-VEGFA-VEGFR2 signaling pathway

**Figure 4:**
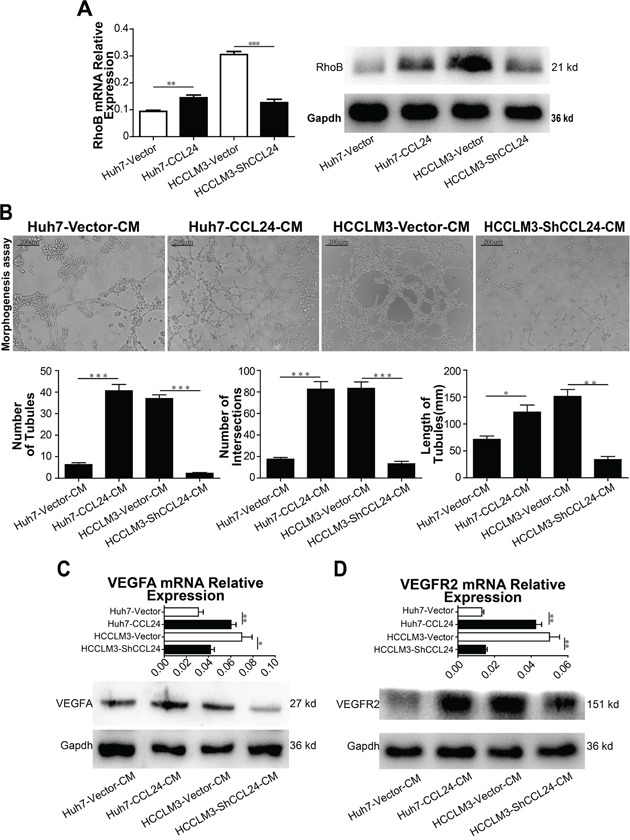
CCL24 promotes migration and invasion of HUVECs and VEGFA signaling pathway **A**. qRT-PCR, western blot analysis of RhoB expression in transfected cells. **B**. Tube formation assay was used to determine the ability of HUVECs to form capillaries after adding different conditioned mediums, and analyzed number of tubules, number of intersections, and length of tubules of different groups. Scale bar, 100×, 200 um. **C-D**. qRT-PCR, western blot analysis of VEGFA, VEGFR2 expression in transfected HCCs. Data shown were means (±SD) from three independent experiments. *P< 0.05, **P< 0.01, ***P<0.001.

The mRNA and protein expression levels of CCR3, which is downstream of CCL24, were not decreased by CCL24 interference (Supplementary Figure S3A). We further observed whether CCL24 was stimulated by Th2 cell factors (IL-4, IL-10 and IL-13) in HCCs or not, qRT-PCR results showed the mRNA level of CCL24 failed to alter after stimulation of Th2 cell factors (Supplementary Figure S3B). RhoB, however, was consistent with the change of CCL24 at both mRNA and protein level (p=0.0092, p=0.0004; Figure 4A), other Rho family members including RhoA, RhoC, CDC42, and MDIA1 had no consistent variation with CCL24 in mRNA and protein levels (Supplementary Figure S3C, S3D). Subsequent qRT-PCR confirmed RhoB was not influenced by Th2 cell factors in HCCs (Supplementary Figure S3E). These negative outcomes appeared to expound one conclusion that CCL24 and RhoB were not regulated by Th2 cell factors in HCC.

Since CCL24 was investigated to accelerate neovascularization in age-related macular degeneration (AMD) [18], we explored the effect of CCL24 on tube formation. Results indicated CCL24 supernatant contributed to Human Umbilical Vein Endothelial Cells (HUVECs) tube formation. Specifically, the number of tubules, the number of intersections, and the length of tubules from the Huh7-CCL24 supernatant stimulation were higher than those from the Huh7-Vector conditioned medium (40.7±2.9 vs. 6.3±0.9, p=0.0003; 82.7±6.9 vs. 17.7±1.5, p=0.0008; 122.0±13.3 vs. 71.7±6.0, p=0.0262; Figure 4B). A similar trend occurred in the HCCLM3-Vector group compared to the HCCLM3-ShCCL24 group (37.0±1.7 vs. 2.3±0.3, p<0.0001; 83.3±5.9 vs. 13.3±2.2, p=0.0004; 151.3±12.7 vs. 33.7±5.9, p=0.0011; Figure 4B).

Interestingly, the migration and invasion ability of HUVECs changed regularly after different HCC conditioned medium inference. Results showed the migration and invasion of the Huh7-CCL24 group were higher than those of the Huh7-Vector group (651.3±18.2 vs. 267.3±13.4, p<0.0001; 384.7±22.2 vs. 101.3±10.7, p=0.0003; Supplementary Figure S4A), and the migration and invasion of the HCCLM3-Vector group were also higher than those of the HCCLM3-ShCCL24 group (696.3±22.8 vs. 279.7±20.5, p=0.0002; 312.0±25.3 vs. 130.7±17.4, p=0.0041; Supplementary Figure S4A). Meanwhile, the addition of CCL24 IgG/BSA to HUVECs further illustrated that CCL24 accelerated the migration and invasion of HUVECs (Supplementary Figure S4B). Moreover, the association of HUVECs with CCL24 IgG was also dose-dependent and sensitive; the recruitment role of HUVECs was apparent when CCL24 IgG concentration was 5 ng/ml (320.0±26.0 vs. 132.7±12.4, p=0.0029; 65.3±10.1 vs. 14.3±3.5, p=0.0088; Supplementary Figure S4B).

**Figure 5:**
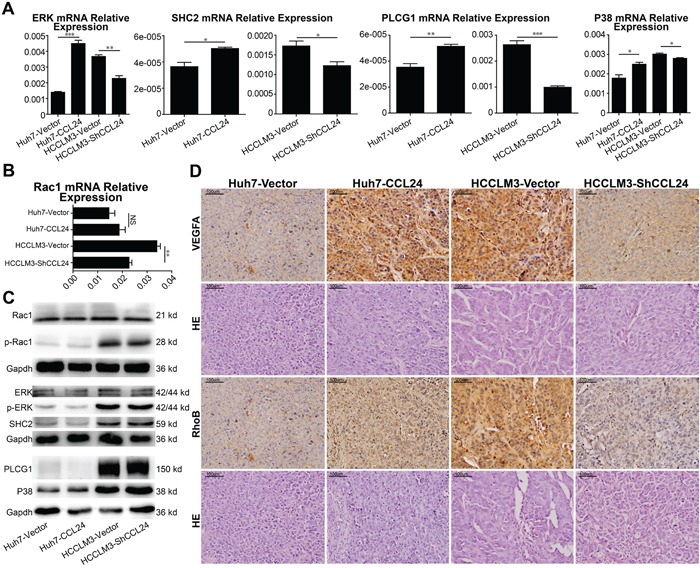
Mechanism of CCL24 promotes HCC cells migration, invasion, and HUVECs angiogenesis via RhoB **A, B**. qRT-PCR analysis of ERK, SHC2, PLCG1, P38, Rac1 expression in transfected cells. **C**. Western blot analysis of Rac1, p-Rac1, ERK, p-ERK, SHC2, PLCG1, P38 expression in transfected cells. **D**. Representative images from tumor serial sections stained with VEGFA, RhoB by immunohistochemistry. Scale bar, 200×, 100 um. Data shown were means (±SD) from three independent experiments. *P< 0.05, **P< 0.01, ***P<0.001.

Based on above results, we analyzed factors known to participate in neovascularization. qRT-PCR results revealed PIGF and VEGFA were in consistent with the change of CCL24 in HCCs (p=0.0457, p=0.0063, Supplementary Figure S4C; p=0.0096, p=0.0472, Figure 4C). To determine the precise receptor of VEGFA, we analyzed the mRNA levels of NRP1, NRP2, and VEGFR2 and showed that only VEGFR2 was in consistent with the change in CCL24 in HCC cells (p=0.001, p=0.0034, Figure 4D; Supplementary Figure S4D). Western blot analysis showed that the VEGFA and VEGFR2 proteins were in accordance with the change in CCL24 in HCC cells (Figure 4C; Figure 4D). There are many pathways downstream of VEGFR2 [19], and we analyzed eight associated main factors to determine the downstream molecular. The results showed that mRNA expression of five factors (ERK, SHC2, PLCG1, P38 and Rac1) were of significance after CCL24 inference (Figure 5A, 5B), but the protein expression of them were not significant after CCL24 inference (Figure 5C). Other factors (VRAP, FAK and AKT) seemed irrelevant with CCL24 in HCCs (Supplementary Figure S5A, S5B). Moreover, VEGFA, RhoB, and CD31 expression levels were in concert with CCL24 as measured by immunohistochemistry of xenograft tumors (Figure 5D; Supplementary Figure S5C).

### RhoB and VEGFA blockade reversed the effect of CCL24

**Figure 6:**
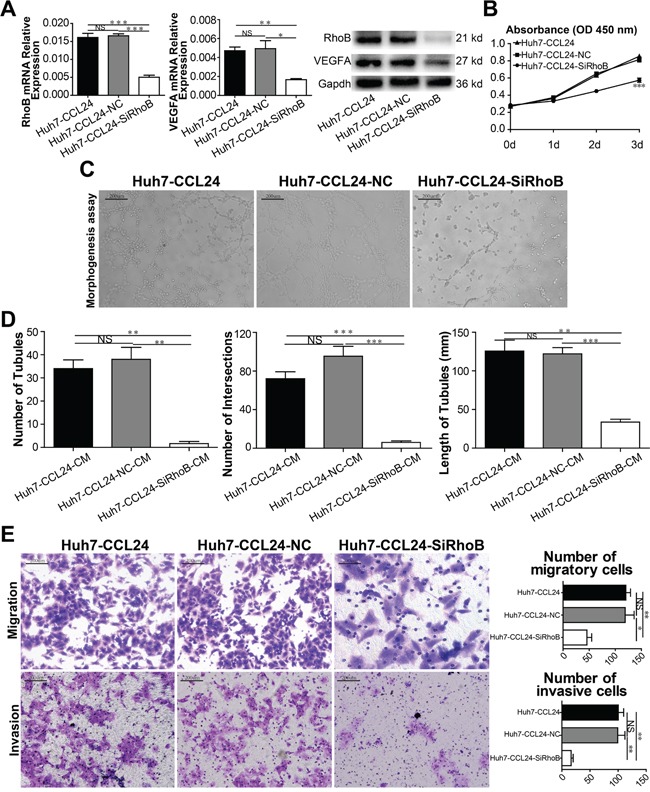
Characterization of siRhoB expression, proliferation, migration, and invasion ability in HCC cells and angiogenesis in HUVECs **A**. qRT-PCR, western blot confirmed RhoB reduced in instant transfected parent cells, and influenced the expression of VEGFA. **B**. Cell proliferation was detected by CCK8 assay. **C-D**. Tube formation assay was used to determine the ability of HUVECs to form capillaries after adding different conditioned mediums, and analyzed number of tubules, number of intersections, and length of tubules of different groups. Scale bar, 100×, 200 um. **E**. The migration and invasion of HCCs were measured by transwell assays after siRhoB interference. Scale bar, 100×, 200 um. Data shown were means (±SD) from three independent experiments. *P< 0.05, **P< 0.01, ***P<0.001.

While RhoB may participate in CCL24-mediated malignancy, it was unclear whether RhoB would play a role in the biological behavior of HCC cells or the expression of VEGFA. Therefore, we knocked down RhoB in Huh7-CCL24 and HCCLM3 using RNA interference. We found that VEGFA was concomitantly varied in line with RhoB (p=0.0001, p=0.0009, Figure 6A; p=0.0009, p=0.0021, Supplementary Figure S6A). Meanwhile, the difference in the cell proliferation of Huh7-CCL24 and Huh7-CCL24-SiRhoB was statistically significant on day 3 (p<0.0001, Figure 6B; the same result was shown in the HCCLM3 group in contrast to the HCCLM3-SiRhoB group, p<0.0001, Supplementary Figure S6B). The role of tube formation in HUVECs by different conditional media of Huh7-CCL24, Huh7-CCL24-NC, Huh7-CCL24-SiRhoB supernatants were also expectedly significant (The P values for the number of tubules, the number of intersections, and the length of tubules were, respectively: p=0.0023, p=0.0011, p=0.001, p=0.0009, p=0.0006, p=0.0032, Figure 6C, 6D; the same finding was also observed in HCCLM3 cells: p=0.0016, p=0.0269, p=0.001, p=0.0045, p=0.0005, p=0.0001, Supplementary Figure S6C, S6D). In addition, the migration and invasion ability of Huh7-CCL24, Huh7-CCL24-NC, and Huh7-CCL24-SiRhoB were also statistically significant (The P values for migration and invasion were p=0.0196, p=0.0044, p=0.0034, p=0.0012, respectively. Figure 6E; the same results were observed in HCCLM3 cells: p=0.0008, p=0.0011, p=0.0138, p=0.0428, respectively. Supplementary Figure S6E).

To further explore the function of VEGFA, we knocked down VEGFA in Huh7-CCL24 and HCCLM3 using RNA interference as well. Result of mRNA level and protein of VEGFR2 were down-regulated after VEGFA RNA interference (Supplementary Figure S7A, SB; Supplementary Figure S8A, S8B). Meanwhile, similar results of proliferation, migration, invasion of HCCs appeared in subsequent experiments (Supplementary Figure S7C, S7D; Supplementary Figure S8C, S8D). To the contrary, RhoB was not concomitantly varied in line with VEGFA (Supplementary Figure S7A, S7B; Supplementary Figure S8A, S8B); maybe correlative phosphorylated indexes needed to be investigated in further research.

## DISCUSSION

In our study, we compared the expression of CCL24 in HCC tissues with adjacent normal tissues firstly, the results showed CCL24 was highly expressed in HCC tissues; further analysis of correlation between CCL24 and prognosis of HCC patients reflected overexpression of CCL24 in HCC predicted shorter OS and higher recurrence rates, which implicated CCL24 as a marker for HCC aggressiveness and a predictor for HCC survival. These findings were consistent with one study in colorectal cancer [14]. In addition, based on depletion and overexpression experiments *in vitro* and *in vivo*, one hypothesis that CCL24 could regulate HCC invasiveness and metastasis was proved; these phenomena naturally urged us to speculate whether CCR3, the downstream of CCL24 changed consistently with CCL24, however, the result seemed CCR3 was irrelevant with CCL24 in HCC in our study. Review CCL24 research in allergy, some study invariably involved Th2 cell factors IL-4 or/and IL-13. Like in asthma, Th2 cells intensively responded to allergens, its excess secretion IL-4, IL-13 promoted recruitment of eosinophils to pulmonary or/and bronchial surface, and subsequent CCL24 produced by eosinophils enhanced above process [20]; similar acceleration of CCL24 by IL-13 stimulation could be found in oesophageal *in vitro* experiment [10]. Meanwhile, via up-regulating CCL24 stimulated by Th2 cell factors, the immune microenvironment was more suitable for colorectal cancer cells to plant or/and progress [14, 15, 20]. So we explored the relevance of CCL24 and IL-4, IL-10, IL-13 in HCCs; unfortunately, negative outcomes pointed that Th2 cell factors failed to modulate CCL24 mRNA level in HCCs, this might attributed to unscientific simulation for immune microenvironment via *in vitro* experiment, or we hadn't found the real causes. Actually, in desensitization of the inflammatory reaction and pathogenesis of asthma, GRK5 and JAK2 produced huge function on promoting CCL24 expression in eosinophil-airway epithelium interaction [21, 22], we tried to investigate both of them expression in HCCs, results revealed they were not associated with CCL24 in HCC (Supplementary Figure S9).

Return to IL-4 modulating the expression of CCL24 in allergy, many study focused the relationship of Rho GTPase family and Th2 cell factors [23–25]. The former (Rho GTPase family) contains 20 members subdivided into 6 subfamilies, including Rho (RhoA/B/C), Rac (Rac1/2/3 and RhoG), CDC42 (CDC42/G25K, TCL, TC10, CHP and WRCH), etc [23]. RhoA had been reported to inhibit Th2 cell differentiation via impairing glycolysis in activated T cells and Th2 cells, then deteriorated allergic airway inflammation through up-relating IL-4 receptor mRNA expression [25]. And the relationship between Rho GTPase family and tumor was also intricate, recent study revealed one of Rho-family effectors MDIA1 could influence the nanomechanical signature of liver cancer and further modulated the biological behavior of HCC [26]. So one hypothesis whether Rho GTPase family directly modulated the expression of CCL24 in HCC bypassing Th2 cell factors to exert corresponding function urged us to learn about the correlation between Rho GTPase family and CCL24. Subsequent experiments testified our speculation that one of Rho GTPase family RhoB changed consistently with CCL24 in HCCs, certainly we couldn't interpret why Th2 cell factors failed to influence RhoB expression in HCC, maybe some unknown complex of immune microenvironment need further exploration in future.

Subsequent experiment phenomena in which CCL24 supernatant contributed to HUVECs tube formation reminded us to focus on neovascularization. As one of classic materials studied for many years in neovascularization, HUVECs had been surveyed in many tumors before; Bais C found VEGF receptor-2 modulated the trafficing of Kaposi's sarcoma to HUVECs [27], and Lin L found CCL18 exposure activated ERK and Akt/GSK-3β/Snail signaling in HUVECs then promoted angiogenesis in breast cancer [28]. At the same time, neovascularization was rarely avoided in HCC, and many research gradually uncovered a link between CCL24 and VEGFA must be existed. One study reported CCL24 and VEGFA synchronously increased in 370 genes analysis of atopic dermatitis disease, one kind of chronic inflammatory skin disease [29]; whereas patients with myalgic encephalomyelitis/chronic fatigue syndrome appeared opposite outcome, VEGFA was reduced along with increases of CCL24 concentrations in plasma levels [30]; and In AMD disease, Takeda found the cause of choroidal neovascular endothelial cells drove from the trafficking of CCL24 to its downstream CCR3 [18].

While in our study, we found that PIGF and VEGFA were consistent with CCL24, and this finding might unify the phenomena of biological behavior of HCCs and HUVECs tube formation. Subsequent experiment we conducted was to disclose the potential signaling pathway. Among many downstream factors of VEGFA [31, 32], we found it was VEGFR2 rather than NRP1 and NRP2 associated with VEGFA in our study. Although some mRNA level of ERK, SHC2, PLCG1, P38, RAC1 which were different pathways of VEGFR2 were consistent with CCL24 in HCCs, their protein expression failed to exhibit difference with each other in HCCs, let alone VRAP, FAK, and AKT which were irrelevant with CCL24 in both mRNA level and protein expression. Incontestably, signaling pathway was not one way to account for neovascularization. Tumor angiogenesis was comprehensive than physical condition when referring to immune microenvironment. Just like preceding discussion, Rho GTPase family might exert huge contribution in our study. Review some articles about Rho GTPase family and angiogenesis, one study disclosed the specific mechanism of VEGF modulating VEGFR2 depended on activation of small GTPase RhoA in the process of angiogenesis [33]. Coincidentally, in Gerald's study, the loss of RhoB in null mice was found to decrease pathological angiogenesis in the ischemic retina and reduce angiogenesis in response to cutaneous wounding via regulating VEZF1-mediated transcription [34]. In addition, RhoB influenced the expression of the matrix metalloproteinases (MMPs) in prostate cancer DU145 and promoted DU145 cell motility and invasion [35]. Regarding RhoC, Hoeppner illustrated that RhoC could stimulate the proliferation of HUVECs by stabilizing nuclear β-catenin, which promotes the transcription of cyclin D1 and subsequently drives cell cycle progression [36]; In one analysis of cDNA microarrays of HCC, Okabe found that RhoC was differentially expressed in hepatitis B virus-positive HCC patients and in hepatitis C virus-positive HCC patients [37], and it modulated the cancer-related function relying on the N terminus of MDIA1 [38]. Certainly, on the basis of the similar structures of RhoA/B/C, they may paly similar biological roles in the immune system by dominating MDIA1 [26].

In our experiment, prior results revealed more invasive cell line HCCLM3-Vector prsented higher RhoA/RhoC levels than Huh7-Vector, however, their correlation with CCL24 was not significant. In contrast, the variation of RhoB shared a similar trend with CCL24. Meanwhile, we found a similar trend of VEGFA and RhoB in xenograft tumors that was in consistent with CCL24. To illustrate the relationship between RhoB and VEGFA, we used small interfering RNA in subsequent experiment to decrease the expression of RhoB/VEGFA, and investigated the transformation of their partner. We detected that siRhoB/siVEGFA blocked the proliferation, migration, and invasion of HCC cells and which were upregulated by CCL24, and western blot revealed RhoB could modulate VEGFA as its upstream. Certainly, about RhoB was not coincided with siVEGFA in HCC, we speculated that other experiments of phosphorylation needed to be explored, for RhoB-induced phosphorylation of myosin light chain on Ser19 after hypoxia stimulation could regulate pulmonary vascular tone and structure [39]. These results showed that CCL24-RhoB-VEGFA-VEGFR2 may participate in HCC malignancy.

Our study has several limitations. On one hand, we mainly focused on the efficiency of CCL24 in tumor cells and ignored the whole immune microenvironment of HCC. On the other hand, we could not fully explain the causes of insignificant expression of CCR3. The specific factors participating in CCL24-RhoB should be further elucidated in our future research. Finally, clinical drawbacks in our study such as the application of radiofrequency ablation (RFA), transcatheter arterial chemoembolization (TACE), percutaneous ethanol injection (PEI), or external radiotherapy for patients with HCC recurrence might influence the OS results.

In conclusion, CCL24 contributed to the malignant biological behavior of HCC through the RhoB-VEGFA-VEGFR2 pathway and was inversely correlated with worse prognosis. This new discovery illustrated the complexity of archenteric cancers and the potential homogeneity of HCC in embryology. Meanwhile, this was the first study to investigate the role of CCL24 in HCC, which will shed light on the diagnosis and target therapy of HCC.

## MATERIALS AND METHODS

### Cell lines and animals

The seven HCC cell lines, normal liver cell line L02, human umbilical vein endothelial cells, and nude mice used in this study are described in the Supporting Information.

### Patients and follow-up

Two independent cohorts consisting of 315 HCC patients and 20 paired fresh cancer/adjacent normal tissue samples were included in this study. The specific information and follow-up process are described in the Supporting Information.

### Statistical analysis

All data were analyzed with SPSS 19.0 software (SPSS Inc., Chicago, IL). Pearson χ^2^ test or Fisher's exact test was adopted to compare qualitative variables. t test or Pearson's correlation test was used to analyze quantitative variables. Kaplan–Meier analysis was used to analyze survival. Survival curves between the different groups were calculated using a log-rank test. Cox proportional hazards model was adopted to determine the univariate or multivariate hazards. A *P* value of <0.05 was considered statistically significant.

For other descriptions of the materials and methods used in this study, see the Supporting Information.

## SUPPLEMENTARY MATERIALS AND METHODS FIGURES AND TABLES


