# Supplementary material for: CCL24 contributes to HCC malignancy via RhoB- VEGFA-VEGFR2 angiogenesis pathway and indicates poor prognosis
Source: Oncotarget. 2016 Nov 24;8(3):5135–48. doi: 10.18632/oncotarget.14095 (PMC5354897; doi:10.18632/oncotarget.14095)
Supplement: Supplementary file 1 [file oncotarget-08-5135-s001.pdf]

CCL24 contributes to HCC malignancy via RhoB- VEGFA-VEGFR2 angiogenesis pathway and indicates poor prognosis

SUPPLEMENTARY MATERIALS AND METHODS

Cell lines and animals

Seven HCC cell lines (Hep3B, HepG2, SMMC-7721, Huh7, MHCC-97L, MHCC-97H, HCCLM3), one normal liver cell (L02), Human umbilical vein endothelial cells (HUVECs; American Type Culture Collection) cultured in high-glucose Dulbecco's modified Eagle medium (DMEM; Invitrogen) (except for SMMC-7721) or Roswell Park Memorial Institute-1640 (RPMI-1640; Invitrogen) (only for SMMC-7721) supplemented with 10% fetal bovine serum (FBS; Invitrogen), 100U/ml penicilin, and 100mg/ml streptomycin (Sigma-Aldrich) at 37°C in a 5% CO2 humidified incubator. Athymic-male BALB/c nude mice (8 weeks old; Shanghai Institute of Material Medicine, Chinese Academy of Science, Shanghai, China) were raised under specific pathogen-free conditions. Animal care and experimental operations were in consistence with the guidelines established by the Shanghai Medical Experimental Animal Care Commission.

Patients and follow-up

20 paired fresh cancer and adjacent cancer tissue samples using for quantitative real-time PCR (qRT-PCR) analysis, were randomly collected from HCC patients who accepted hepatic resection with informed agreement according to the ethical standards from 2009 to 2010 in Liver Cancer Institute, Zhongshan Hospital, Fudan University, Shanghai, China. With approvement by institutional review board of Liver Cancer Institute based on the Declaration of Helsinki, national and international guidelines, all tissues were collected immediately once the hepatic tumor was removed in operation theater and transported in liquid nitrogen.

Cell transfection

Lentivirus CCL24, ShCCL24, blank vector lentiviral were purchased from GenePharma (Shanghai, China) and were transfected into Huh7 and HCCLM3 cells according to the manufacturer's instructions. Stable knockdown and over-expression clones were confirmed by qRT-PCR and Enzyme-Linked Imunosorbent Assay (ELISA). The shRNA target sequences were as follows:

CCL24-ShRNA: GCCTGATGACCATAGTAACCA

RhoB small interfering RNA (RhoB siRNA) and the negative control (NC) sequences were purchased from GenePharma (Shanghai, China). Huh7-CCL24 and HCCLM3 cell lines were transiently transfected with RhoB siRNA and NC using the Lipofectamine RNAi MAX transfection reagent (Invitrogen, Carlsbad, CA, USA) based on the manufacturer's instructions. These cells were collected after incubating for 48h for further analysis. And the efficiency of transfection were confirmed by qRT-PCR and Western blot. The siRNA sequences are as follows:

RhoB-SiRNA: GCUGAUCGUGUUCAGUAAGTT

VEGFA small interfering RNA (VEGFA siRNA) and the negative control (Control) sequences were purchased from GenePharma (Shanghai, China). Huh7-CCL24 and HCCLM3 cell lines were transiently transfected with VEGFA siRNA and Control using the Lipofectamine RNAi MAX transfection reagent (Invitrogen, Carlsbad, CA, USA) based on the manufacturer's instructions. These cells were collected after incubating for 48h for further analysis. And the efficiency of transfection were confirmed by qRT-PCR and Western blot. The siRNA sequences are as follows:

VEGFA-SiRNA: GCAUUGGAGCCUUGCCUUG CUTT.

Cell proliferation, Matrigel migration and invasion assays

Cells were seeded into 96-well plates in 100μL aliquots. At the designed time points, 10μL of CCK-8 solution (Dojindo, Japan) was added to the cells, then plates were incubated for subsequent 2h. The absorbance was measured at 450nm to determine the numbers of viable cells in each well. All experiments were performed in triplicate for three days. For Matrigel migration and invasion assays, cell culture was performed in Transwell chambers (8μm for Huh7 and HCCLM3, 24-well format; Corning, USA). The insert membranes were coated with diluted Matrigel for the invasion assay (BD Biosciences, USA). Cells (1×10^5) were then added to the upper chamber and cultured for 48h. The insert membranes were not coated with Matrigel for the migration assay, and the cells were cultured under the same conditions. Finally, the insert membranes were cut and stained with crystal violet (0.04% in water; 1 ml), and the permeating

cells were counted under an inverted microscope. Another migration assay was examined *in vitro* using Transwell chambers (3μm for HUVECs, 24-well format; Corning, USA) as the manufacturer's instructions. HUVECs were serum deprived for 24h, then 2.5 × 10^4 cells which were suspended were seeded in triplicate on Matrigel-coated (Matrigel : DMEM=1:1) inserts and shifted to chambers containing different conditioned medium based on aim of experiment and incubated at 37°C for 24h. Different CM(conditioned medium) were harvested from Huh7-Vector, Huh7-CCL24, HCCLM3-Vector, HCCLM3-ShCCL24, Huh7-Vector+BSA (5/10/50ng/ml), Huh7-Vector+CCL24 (5/10/50ng/ml), HCCLM3-ShCCL24+BSA, HCCLM3-ShCCL24+CCL24 (5/10/50ng/ml), HUVEC+BSA, HUVEC+CCL24 (5/10/50ng/ml), Huh7-CCL24-NC, Huh7-CCL24-SiRhoB, HCCLM3, HCCLM3-NC, HCCLM3-SiRhoB, into lower transwell chambers. Four fields were randomly chosen and counted for each membrane. All experiments were performed in triplicate.

Th2 cell factors stimulation

Huh7-Vector, Huh7-CCL24, HCCLM3-Vector and HCCLM3-ShCCL24 (10^6) were plated in 3mL DMEM, 10% fetal bovine serum in four-well plates. After 24h at 37°C, fresh DMEM containing 10% fetal bovine serum, 10 ng/mL of IL-4, IL-10, and IL-13 (from R&D Systems) were added in 0.5mL cells were cultured for a further 24h, respectively. RNA was isolated by Trizol Reagent (Invitrogen, Carlsbad, CA, USA), subsequent qRT- PCR for CCL24 was done.

Endothelial tube formation assay

Endothelial tube formation assay kit (Cell Biolabs, SanDiego, CA, USA) was used to assess Angiogenesis *in vitro*. 96-well culture plates were frozen at 4°C in refrigerator in advance, then each well was coated with a thin layer of extracellular matrix gel 50 μl/well getting from Engelbreth-Holm-Swarm tumor cells. Then 96-well culture plates shifted to 37°C for 1h, where it would polymerize. HUVECs were suspended in the supernatants obtained from Huh7-Vector, Huh7-CCL24, HCCLM3-Vector, HCCLM3-ShCCL24 cells. Then HUVECs (1.5 × 10^4 cells/well) were cultured in the polymerized extracellular matrix gel in 150μL of serum-free medium. The tubule numbers were assessed under a light microscope after 14h of incubation at 37°C with 5% CO2.

qRT-PCR

Total RNA of different cells was isolated depending on Trizol Reagent (Invitrogen, Carlsbad, CA, USA) and then decreased amplification of genomic DNA through use of DNase. Each one microgram of total mRNA was reversely transcribed into complementary DNA (cDNA) using SuperScript III Reverse Transcriptase (Invitrogen) according to the manufacturer's instructions. Then Quantitative real-time PCR was conducted on an ABI 7900 HT instrument (Applied Biosystems, Foster, Foster City, CA). The proportion of elements for the qRT-PCR was refered to SYBR PrimeScript RT-PCR Kit (Takara, Japan), and GAPDH was used as an internal control. Relative mRNA levels were calculated based on the Ct values, which were corrected for GAPDH expression according to the following equation: $2^{-\Delta\Delta Ct}$ [ΔCt = Ct (CCL24) – Ct (Gapdh)]. All experiments were conducted in triplicate. The primers of CCL24, VEGFA, VEGFR2, ERK, SHC2, PLCG1, P38, Rac1, JAK2, GRK5, PIGF, CCR3, NRP1, NRP, CDC42, MDIA1, RhoA, RhoB, RhoC, VRAP, FAK, AKT and Gapdh are shown in Supplementary Table S1.

Western blot

Whole-cell lysates were collected with RIPA lysis buffer (Beyotime, China) mixing protease inhibitor cocktail (Sigma-Aldrich, St. Louis, MO). The cytosol and nuclear extracts were prepared by using the nuclear extract kit (Active Motif, Carlsbad, CA). Protein samples were separated by sodium dodecyl sulfate-polyacrylamide gel electrophoresis (SDS-PAGE), and electrotransferred onto polyvinylidene difluoride (PVDF) membranes (Millipore, Bedford, MA, USA). Then membranes were blocked with 5% BSA, washed, and incubated with primary antibodies at 4°C overnight in refrigrater. After returning to room temperature for 1h, washed, then membrane was incubated with horseradish peroxidase (HRP)-conjugated secondary antibodies (Santa Cruz Biotechnology) for 1h and detected using enhanced chemiluminescence method (Pierce, Rockford, IL, USA). Then observed by fluorescence microscopy (Leica Microsystems Imaging Solutions, Cambridge, UK). The primary antibodies of VEGFA, VEGFR2, RhoB, Rac1, p-Rac1, ERK, p-ERK, SHC2, PLCG1, P38, CCR3, MDIA1, RhoA, RhoC, CDC42, AKT, Gapdh were shown in Supplementary Table S2.

Enzyme-linked imunosorbent assay (ELISA)

The supernatant from L02, Hep3B, HepG2, SMMC-7721, Huh7, MHCC-97L, MHCC-97H, HCCLM3, Huh7-Vector, Huh7-CCL24, HCCLM3-Vector and HCCLM3-ShCCL24 cultured in prepared 96-wells in human CCL24 ELISA kits (R&D Systems) based on its specifications. All experiments were performed in triplicate.

TMA and immunohistochemistry

Tissue microarray (TMA) was acquired as previously described (1). All of HCC samples were

reviewed by two histopathologists. We randomly collected patient specimens from TMA (training cohort, n = 359; validation cohort, n = 362), and then TMA were constructed by Shanghai Biochip Co, Ltd. Briefly, after rehydration and microwave antigen retrieval, primary antibodies were applied to the slides for incubating at 4 °C overnight, followed by secondary antibody incubation (GK500705, Gene Tech, China) at room temperature for 1h after taking out from 4 °C and staying in PBS for a while. Then washed, stained with DAB, counterstained with hematoxylin. Negative controls using PBS instead of primary antibody were included in all assays. The primary antibodies used in TMA and immunohistochemistry of CCL24, VEGFA, RhoB, and CD31 were shown in Supplementary Table S2.

Evaluation of immunohistochemical variables

Each case was scored by two independent pathologists who didn't know knowledge of the patient characteristics by semi-quantitative method. The staining score was divided into four grades: “0”, negative: no staining; “+”, mild: weak staining in less than one-third of the cells; “++”, intermediate: moderate staining in one-third to two-thirds of the cells; and “+++”, strong: strong staining in more than two-thirds of the cells. CCL24 expression was categorized by staining intensity as low (“0” and “+”) or high (“++” and “+++”). HCC patients with low or high expression were classified as $CCL24^{Low}$ or $CCL24^{High}$, respectively. Duplicate spots for each tumor showed a good level of homogeneity for the staining protocol. The higher score was adopted as the final score in cases with differences between duplicate tissue cores.

In vivo tumorigenesis and metastasis

Huh7-Vector, Huh7-CCL24, HCCLM3-Vector, HCCLM3-ShCCL24, cells (5×10^6 cells) were re-suspended in 100μL serum-free DMEM mixed Matrigel (BD Biosciences, San Jose, CA) (1:1). Then cells were injected subcutaneously into the upper right dorsal region of nude mice. After 4 weeks' injection, when subcutaneous tumors reached approximately 1cm in length, all tumors were peeled, sheared into $1mm^3$ volume and inserted into the livers of 24 different nude mice (n = 6 for each group). Mice were monitored frequently by measuring the weight and width of tumor and were sacrificed under anesthesia after 6 weeks after injection. Then tumor, liver, and lung tissues were peeled, soaked in formalin, and embedded in paraffin or used for other applications. Consecutive sections for each lung tissue block and staining with hematoxylin and eosin for the search of lung metastasis. Tumor volume was measured using the following formula: V = π/6× (larger diameter) × (smaller diameter^2). Lung metastases was authenticated and the number was calculated and evaluated independently by two pathologists. Lung metastases were classified into four grades on the basis of number of HCC cells in the maximal section of the metastatic lesion: grade I, ≤ 20 tumor cells; grade II, 20 to 50 tumor cells; grade III, 50 to 100 tumor cells; and grade IV, >100 tumor cells.

Statistical analysis

All data were analyzed with SPSS 19.0 software (SPSS Inc., Chicago, IL), Pearson X^2 test or Fisher's exact test was used to compare qualitative variables, t test or Pearson's correlation test was used to analyze quantitative variables, Kaplan–Meier analysis was used to judge the survival; survival curves between different groups were calculated with a log-rank test, and cox proportional hazards model forecasted the univariate or multivariate hazards. *P* value <0.05 was considered statistically significant.

Cox's model and how the stepwise variable selection was done

1. Common operation: Seclet “Cox Regression” in SPSS; “Time”: insert OS_Time (choose os group); “Status”: insert OS_Status

2. For Univariate analysis: insert each variate into “Covariates” (like age); “Method”: Enter; Click “OK”; Read the “Sig.”; ultimately record P value.

3. For Multivariate analysis: insert all variates (The “Sig.” of variates must <0.05 via above operation) into “Covariates” (OS group: γ-GT, Tumor numbe, Tumor size, Microvascular invasion TNM stage, BCLC stage, CCL24 level); “Method”: Enter; Click “OK”; Read the “Sig.”; then change “Method”: Backward LR, to validate the result of above outcomes (when choose “Enter”); ultimately record P value.

REFERENCE

1. Liu WR, Tian MX, Yang LX, Lin YL, Jin L, Ding ZB, Shen YH, et al. PKM2 promotes metastasis by recruiting myeloid-derived suppressor cells and indicates poor prognosis for hepatocellular carcinoma. Oncotarget 2015;6:846-861.

SUPPLEMENTARY FIGURES AND TABLES

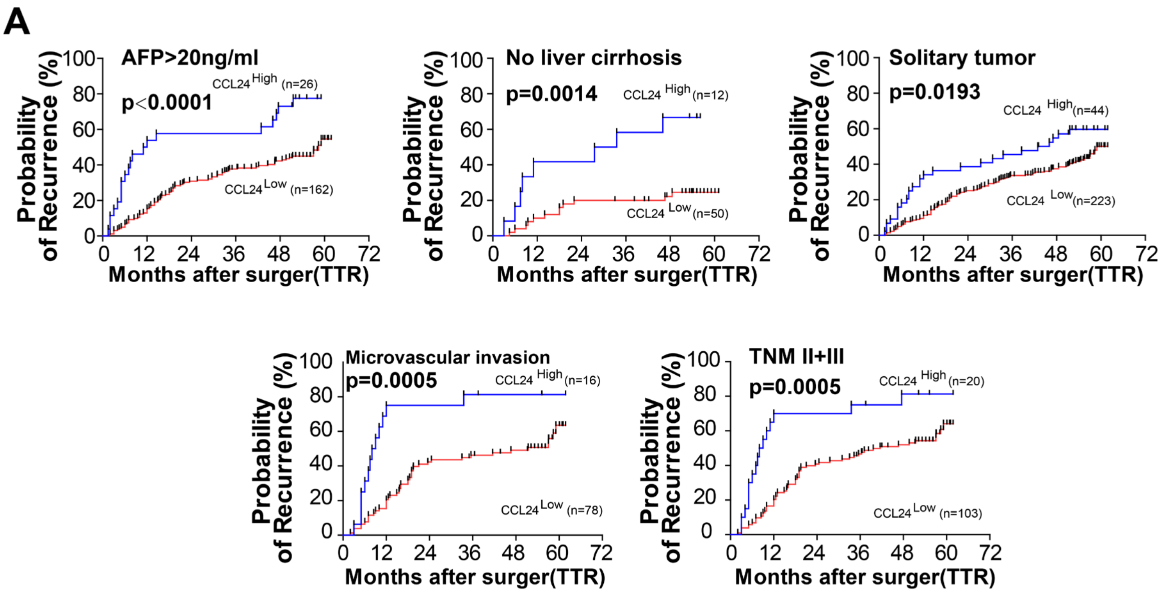

Supplementary Figure S1: CCL24 expression and prognostic value in HCC tissue (n= 315). A. Prognostic role of CCL24 in AFP>20 ng/mL, No liver cirrhosis, Solitary tumor, Microvascular invasion, TNM stage II + III subgroups.

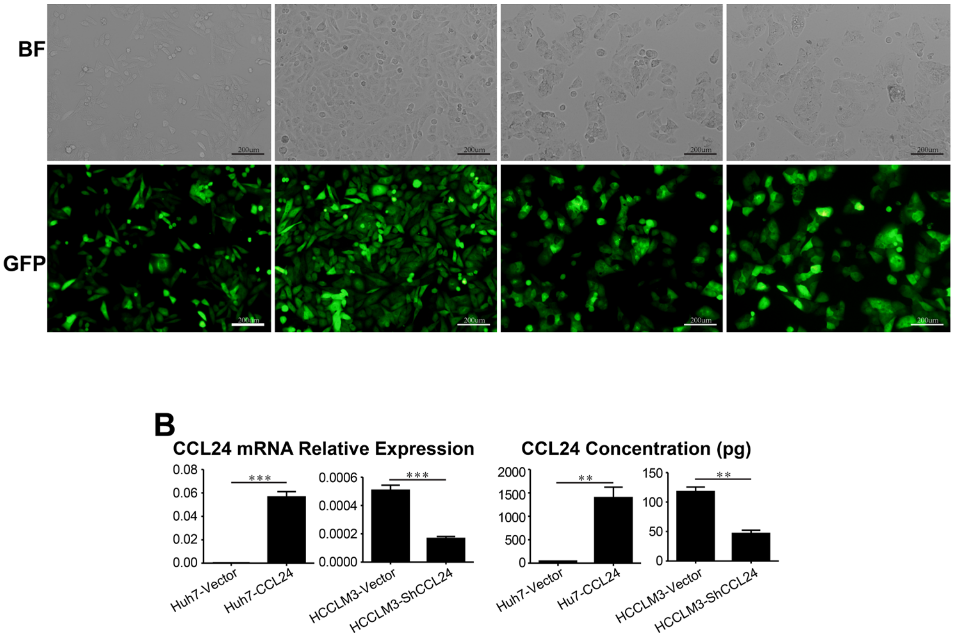

Supplementary Figure S2: Transfection Efficiency of CCL24 Overexpression, Vector and CCL24-ShRNA. A. The efficiency of CCL24 in HCCs. BF: Bright Field, GFP: green fluorescent protein, the lentivirus harbors GFP gene. Amplification: 100×, Scale Bar: 200 um. **B.** qRT-PCR, ELISAs confirmed CCL24 expression in stably transfected and parent cells. Data shown were means (±SD) from three independent experiments. *P< 0.05, **P< 0.01, ***P<0.001.

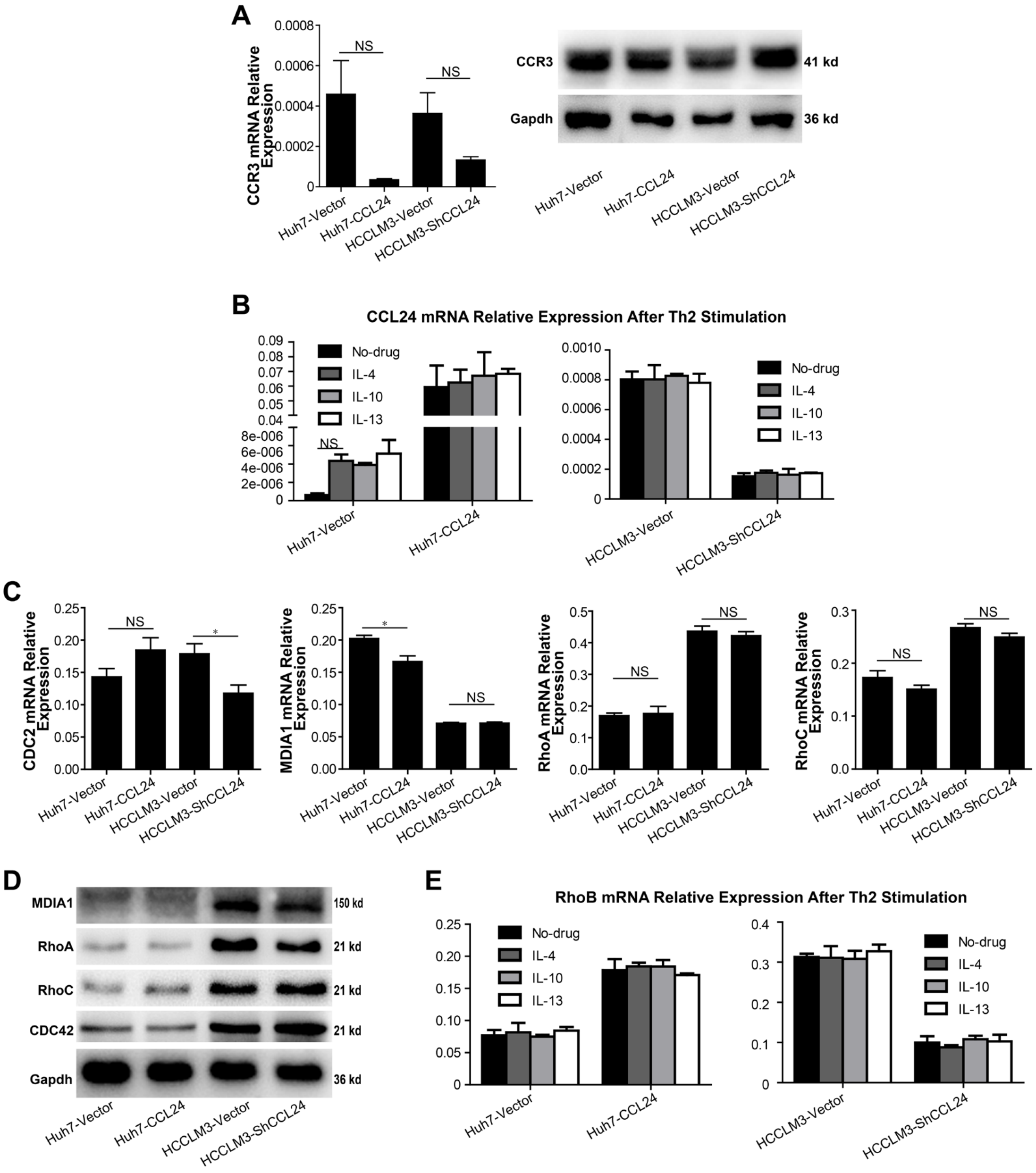

Supplementary Figure S3: Negative outcomes of CCR3 and Th2 cell factors in HCC cells. A. qRT-PCR analysis of CCR3 expression in transfected cells, western blot analysis of CCR3 expression in transfected cells. **B.** qRT-PCR analysis of CCL24 expression in transfected cells after Th2 cell factors stimulation. **C.** qRT-PCR analysis of CCR3, CDC42, MDIA1, RhoA and RhoC expression in transfected cells. **D.** Western blot analysis of CDC42, MDIA1, RhoA and RhoC expression in transfected cells. **E.** qRT-PCR analysis of RhoB expression in transfected cells after Th2 cell factors stimulation. Data shown were means (±SD) from three independent experiments. *P< 0.05, **P< 0.01, ***P<0.001.

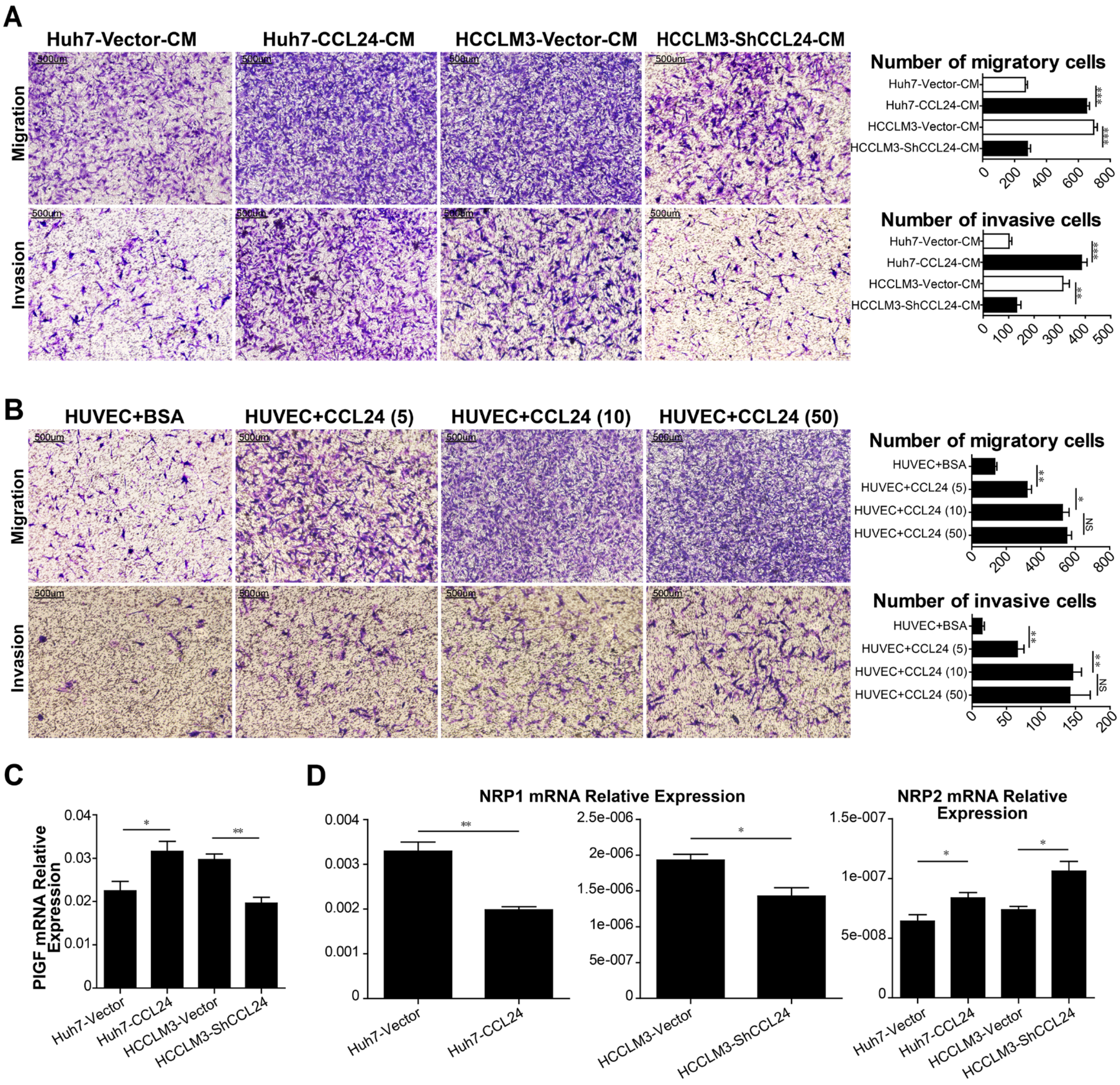

Supplementary Figure S4: CCL24 promotes migration and invasion of HUVECs and VEGFA signaling pathway. A. The migration and invasion of HUVECs were measured by transwell assays after adding different conditioned mediums form transfected HCCs. Scale bar, 40×, 500 um. **B.** The migration and invasion of HUVECs were measured by transwell assays after adding different concentration of CCL24 IgG/BSA (respectively: 5 ng/ml, 10 ng/ml, 50 ng/ml). Scale bar, 40×, 500 um. **C-D.** qRT-PCR analysis of PIGF, NRP1, NRP2, in transfected cells. Data shown were means (±SD) from three independent experiments. *P< 0.05, **P< 0.01, ***P<0.001.

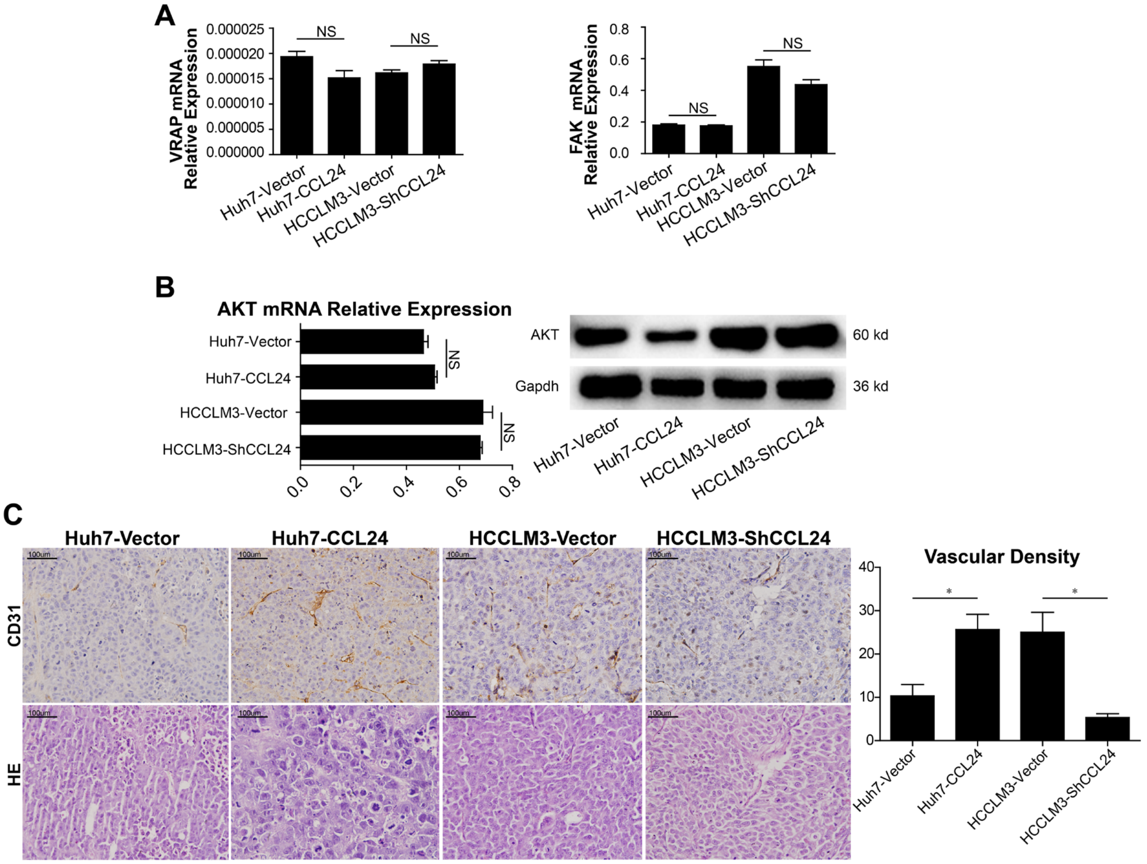

Supplementary Figure S5: Mechanism of CCL24 promotes HCC cells migration, invasion, and HUVECs angiogenesis via RhoB. A-B. qRT-PCR analysis of VRAP, FAK, and AKT expression in transfected cells. B. Western blot analysis of AKT expression in transfected cells. **C.** Representative images from tumor serial sections stained with CD31 by immunohistochemistry. Scale bar, 200×, 100 um. Data shown were means (±SD) from three independent experiments. *P< 0.05, **P< 0.01, ***P<0.001.

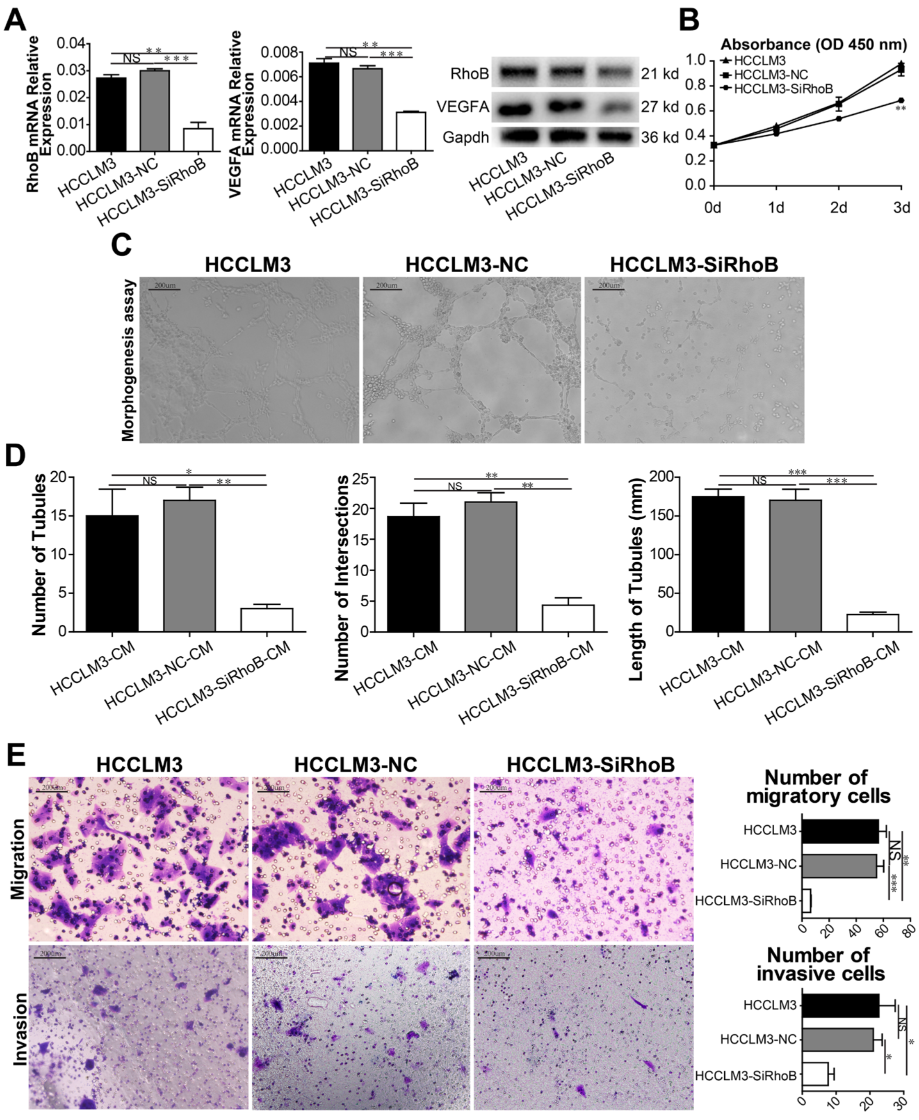

Supplementary Figure S6: Characterization of siRhoB expression, proliferation, migration, and invasion ability in HCC cells and angiogenesis in HUVECs. A. qRT-PCR, western blot confirmed RhoB reduced in instant transfected parent cells, and influenced the expression of VEGFA. B. Cell proliferation was detected by CCK8 assay. **C-D.** Tube formation assay was used to determine the ability of HUVECs to form capillaries after adding different conditioned mediums, and analyzed number of tubules, number of intersections, and length of tubules of different groups. Scale bar, 100×, 200 um. **E.** The migration and invasion of HCCs were measured by transwell assays after siRhoB interference. Scale bar, 100×, 200 um. Data shown were means (±SD) from three independent experiments. *P< 0.05, **P< 0.01, ***P<0.001.

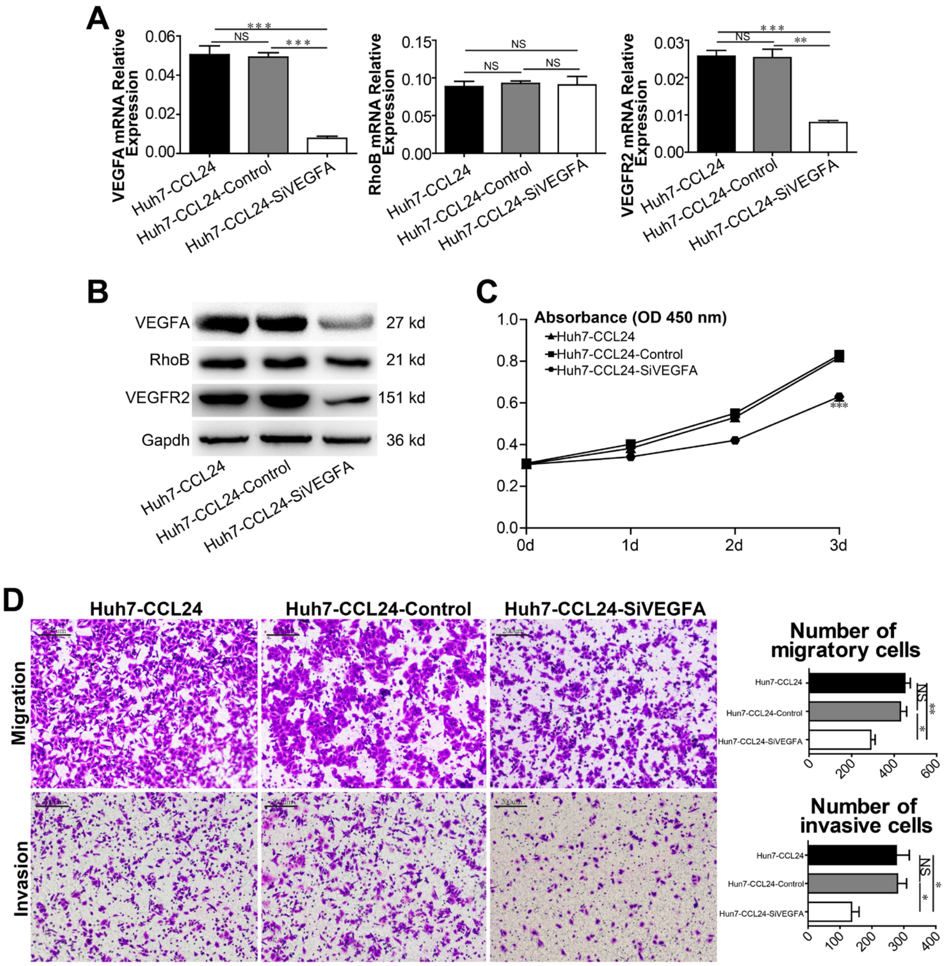

Supplementary Figure S7: Characterization of siVEGFA expression, proliferation, migration, and invasion ability in HCC cells. A-B. qRT-PCR, western blot confirmed VEGFA reduced in instant transfected parent cells, and influenced the expression of RhoB and VEGFR2. **C.** Cell proliferation was detected by CCK8 assay. **D.** The migration and invasion of HCCs were measured by transwell assays after siVEGFA interference. Scale bar, 100×, 200 um. Data shown were means (±SD) from three independent experiments. *P< 0.05, **P< 0.01, ***P<0.001.

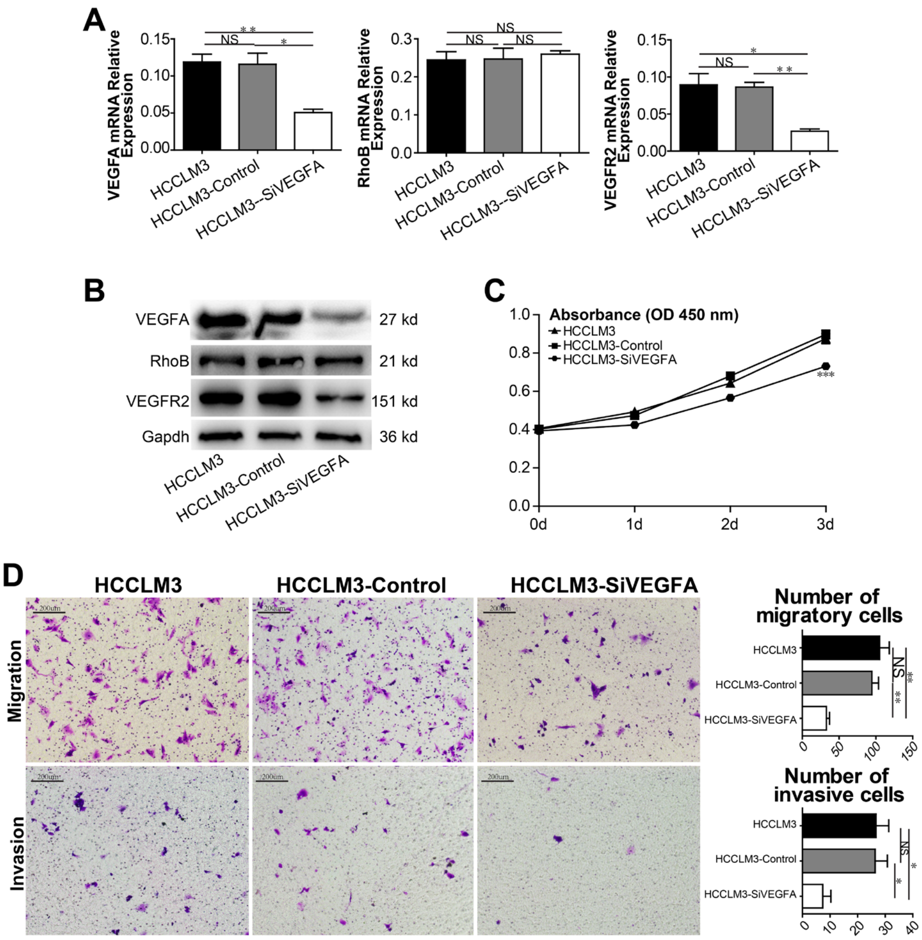

Supplementary Figure S8: Characterization of siVEGFA expression, proliferation, migration, and invasion ability in HCC cells. A-B. qRT-PCR, western blot confirmed VEGFA reduced in instant transfected parent cells, and influenced the expression of RhoB and VEGFR2. **C.** Cell proliferation was detected by CCK8 assay. **D.** The migration and invasion of HCCs were measured by transwell assays after siVEGFA interference. Scale bar, 100×, 200 um. Data shown were means (±SD) from three independent experiments. *P< 0.05, **P< 0.01, ***P<0.001.

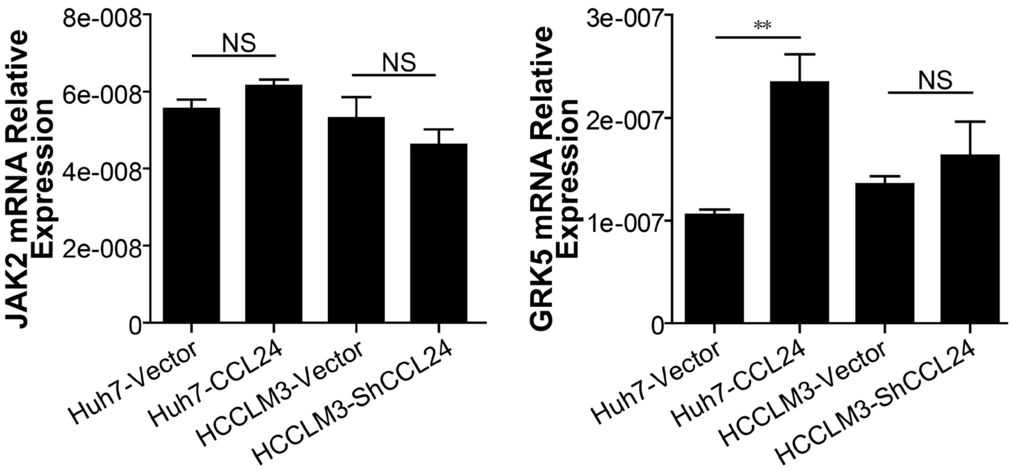

Supplementary Figure S9: Negative outcomes of JAK2 and GRK5 in transfected cells. qRT-PCR analysis of VRAP, FAK, and AKT expression in transfected cells. Data shown were means (±SD) from three independent experiments. *P< 0.05, **P< 0.01, ***P<0.001.

Supplementary Table S1: Sequence of primers for qRT-PCR

Gene	Forward primer (5'–3')	Reverse primer (5'–3')
CCL24	AGCAACACTCTCCAGGGATG	TCAGGAGGAGAAGGCAAAGA
VEGFA	AGGGCAGAATCATCACGAAGT	AGGGTCTCGATTGGATGGCA
VEGFR2	GTGACCAACATGGAGTCGTG	TGCTTCACAGAAGACCATGC
ERK	TCACACAGGGTTCCTGACAGA	ATGCAGCCTACAGACCAAATATC
SHC2	TCCTACGTCGTGCGGTACAT	CCTCATGGAGCCGGTTGATG
PLCG1	GGAAGACCTCACGGGACTTTG	GCGTTTTCAGGCGAAATTCCA
P38	GGGGCAGATCTGAACAACAT	CAGGAGCCCTGTACCACCTA
Rac1	ATGTCCGTGCAAAGTGGTATC	CTCGGATCGCTTCGTCAAACA
JAK2	ATCCACCCAACCATGTCTTCC	ATTCCATGCCGATAGGCTCTG
GRK5	CCAACACGGTCTTGCTGAAAG	TCTCTGTCTATGGTCCTTCGG
PIGF	TGACATGGTTGTGCATCTGTT	ACTCTATCAGTGGTGCTCCATAC
CCR3	TGGCATGTGTAAGCTCCTCTC	CCTGTCGATTGTCAGCAGGATTA
NRP1	CCACAGTGGAACAGGTGATG	GCACGTGATTGTCATGTTCC
NRP2	ATACCACACCAAGGCTGGAG	ACCACCTAGTCCGGGAGAGT
CDC42	TCCCCATCTGGTGCTCTTAG	TGGCAAACAAATGTCCTTGA
MDIA1	AAGGTGAAGGTCGGAGTCAAC	GGGGTCATTGATGGCAACAATA
RhoA	AGCCTGTGGAAAGACATGCTT	TCAAACACTGTGGGCACATAC
RhoB	ATCCCCGAGAAGTGGGTCC	CGAGGTAGTCGTAGGCTTGGA
RhoC	GGAGGTCTACGTCCCTACTGT	CGCAGTCGATCATAGTCTTCC
VRAP	CCTGTCCTACGGAAGAGCTG	CCTGTCCTCTGTCCTGAAGC
FAK	TGGTGAAAGCTGTCATCGAG	TACTCTTGCTGGAGGCTGGT
AKT	TTGAAGGCACTGGGGTAAAG	GAAGGCAGCTGTTTCTTTGG
Gapdh	TGTGGGCATCAATGGATTTGG	ACACCATGTATTCCGGGTCAAT

Supplementary Table S2: Primary antibodies for western blot, IHC

Antibody	Concentration for WB	Concentration for IHC	Specificity	Company
VEGFA	1:1000	1: 100	Rabbit polyclonal	Abcam
VEGFR2	1:1000		Rabbit polyclonal	Abcam
RhoB	1:1000	1:50	Mouse monoclonal	Santa Cruz Biotechnology
Rac1	1:1000		Rabbit Polyclonal	Cell Signaling Technology
p-Rac1	1:1000		Rabbit Polyclonal	Cell Signaling Technology
ERK	1:1000		Rabbit Polyclonal	Cell Signaling Technology
p-ERK	1:2000		Rabbit monoclonal	Cell Signaling Technology
SHC2	1:1000		Rabbit polyclonal	Santa Cruz Biotechnology
PLCG1	1:1000		Mouse monoclonal	Abcam
P38	1:1000		Mouse monoclonal	Abcam
CCR3	1:500		Rabbit monoclonal	Abcam
MDIA1	1:1000		Rabbit Polyclonal	Cell Signaling Technology
RhoA	1:1000		Rabbit monoclonal	Cell Signaling Technology
RhoC	1:1000		Rabbit monoclonal	Cell Signaling Technology
CDC42	1:1000		Rabbit monoclonal	Cell Signaling Technology
AKT	1:1000		Rabbit monoclonal	Cell Signaling Technology
Gapdh	1:2500		Rabbit polyclonal	Abcam
CCL24		1:100	Goat polyclonal	Santa Cruz Biotechnology
CD31		1:50	Rabbit polyclonal	Abcam

Abbreviations: WB, western blot; IHC, immunohistochemistry.
